# Attitudes and experiences of lifestyle healthcare professionals in the care of metabolic and bariatric surgery patients in the Netherlands

**DOI:** 10.1016/j.obpill.2026.100261

**Published:** 2026-03-25

**Authors:** Franshelis K. Garcia, Bob C. Mulder, Annemarie Wagemakers, Eric J. Hazebroek, Kirsten T. Verkooijen

**Affiliations:** a Wageningen University & Research, Social Sciences Group, Chair Group Health and Society, P.O. Box 8130, bode 60, 6700 EW, Wageningen, the Netherlands; b Wageningen University & Research, Social Sciences Group, Chair Group Health and Society, Hollandseweg 1, 6706 KN, Wageningen, the Netherlands; c Wageningen University & Research, Social Sciences Group, Chair Group Strategic Communication, P.O. Box 8130, bode 79, 6700 EW, Wageningen, the Netherlands; d Wageningen University & Research, Social Sciences Group, Chair Group Strategic Communication, Hollandseweg 1, 6706 KN, Wageningen, the Netherlands; e Wageningen University & Research, Agrotechnology and Food Sciences Group, Chair Group Human Nutrition & Health, P.O. Box 9100, bode 62, 6700 EW, Wageningen, the Netherlands; f Wageningen University & Research, Agrotechnology and Food Sciences Group, Chair Group Human Nutrition & Health, Stippeneng 4, 6708WE, Wageningen, the Netherlands; g Department of Bariatric Surgery, Vitalys, Rijnstate Hospital, P.O. 9555, 6800 TA, Arnhem, the Netherlands; h Department of Bariatric Surgery, Vitalys, Rijnstate Hospital, Wagnerlaan 55, 6815 AD, Arnhem, the Netherlands

**Keywords:** Dietician, Lifestyle coach, Lifestyle healthcare professional, Metabolic and bariatric surgery, Physiotherapist, Qualitative study

## Abstract

**Background:**

Lifestyle healthcare professionals (LHCPs), such as dietitians, physiotherapists, and lifestyle coaches, play an important role in supporting patients undergoing metabolic and bariatric surgery (MBS). Preoperatively, they provide nutritional counseling and behavioral support to prepare patients for surgery. Postoperatively, they assist with dietary adaptation and lifestyle changes, supporting long-term weight management and improved outcomes. Despite this critical role, little is known about LHCPs’ attitudes toward MBS, though these views shape care quality, patient engagement, and outcomes. Additionally, research among LHCPs has largely focused on their practical roles, while evidence on their experiences supporting this patient group remains scarce. Hence, this study explores the attitudes and experiences of LHCPs who care for MBS patients.

**Methods:**

This qualitative descriptive study explored the attitudes and experiences of 22 LHCPs, practicing within the Dutch healthcare system, using semi-structured interviews. Data were analyzed inductively and deductively using thematic analysis.

**Results:**

Four main themes emerged: (1) MBS as a tool of last resort, (2) Surgery alone is not enough, (3) The (non) ideal MBS candidate and patient motivation, and (4) Care delivery challenges. LHCPs expressed generally positive attitudes toward MBS, recognizing its effectiveness, but were cautious about the risks, while also acknowledging that long-term success requires sustained behavioral and psychological support. Patient motivation was seen as critical, yet declining adherence to lifestyle changes post-surgery posed challenges. LHCPs reported various challenges in providing post-surgical care, particularly in primary care settings, due to limited expertise and skills, strict care protocols, and insufficient insurance-covered consultation time.

**Conclusion:**

LHCPs see MBS as effective but face care challenges. As patients transition back to primary care, LHCPs become critical points of contact. Strengthening training and support structures for LHCPs is essential to ensure optimal long-term care for MBS patients**.**

## Graphical abstract

**Figure:**
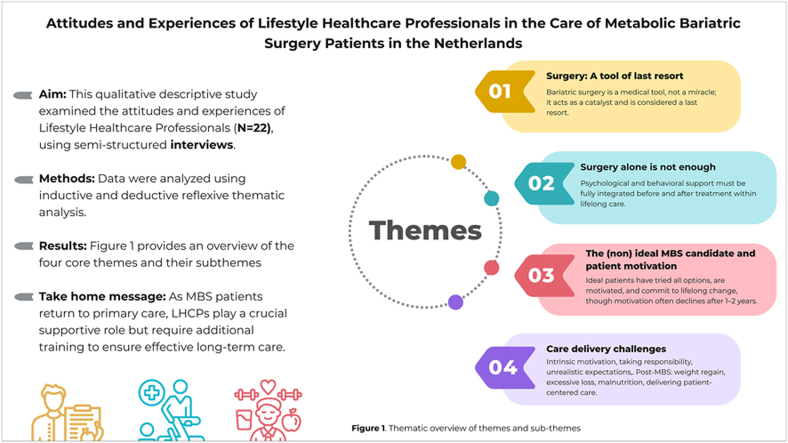

## 1 Introduction

The World Health Organization defines obesity as an abnormal or excessive fat accumulation that impairs health [1]. Obesity is most commonly classified using body mass index (BMI), with severe obesity (Class III) defined by a BMI of ≥40 kg/m^2^. Severe obesity poses a major global and national health challenge and increases the risk of numerous chronic conditions, including cardiovascular disease, type 2 diabetes, and various types of cancer [[1], [2], [3]].

Metabolic and Bariatric surgery (MBS) is currently recognized as the most effective intervention for severe obesity, often resulting in substantial weight loss and improvements in weight-related comorbidities [2,[4], [5], [6], [7], [8]]. However, long-term success depends on patients' lifelong adherence to recommended lifestyle changes [2,3,9,10]. These lifestyle changes not only contribute to the effectiveness of the surgery but also help minimize postoperative complications, such as nutritional deficiencies, gastrointestinal symptoms, loss of lean muscle mass, and in some cases, weight regain [10,11]. Therefore, lifestyle support is important both before and after surgery, as this support is critical for achieving and maintaining long-term success [10,11].

Lifestyle healthcare professionals (LHCPs) - professionals, who support behavior change by addressing nutrition, physical activity, sleep, stress, avoidance of risky substances, and positive social connections [12] - play a central role in supporting MBS patients by helping them build and sustain the required lifestyle changes throughout a patient's surgical journey. For example, during the preoperative phase, dietitians assess dietary habits and nutritional status, provide counseling or education sessions, and may guide patients through a dietary lifestyle program in primary care before surgery to meet guidelines and improve readiness [10,11,13]. LHCPS, such as physiotherapists or lifestyle coaches, conduct fitness assessments and support regular physical activity [10,11,13]. Postoperatively, these professionals continue to provide nutrition, exercise, and behavior-change support, particularly during the first year after surgery. Overall, the engagement of LHCPs throughout the surgical pathway supports sustained behavioral change, promotes long-term weight management, and improves patient health outcomes [3,10,11,13].

Despite LHCPs' central role and importance within the MBS pathway, little is known about their attitudes – referring to their beliefs, feelings, views, and opinions - toward MBS and its patients [14] or their experiences in providing care and support to this patient group. This gap is significant as LHCPs’ attitudes and experiences directly shape the quality of care, patient engagement, and long-term patient outcomes [10,11,13]. To date, most studies focusing on LHCPs have predominantly examined the *practical* aspects of their roles, such as expected responsibilities and routine clinical activities, particularly for dietitians and, to a lesser extent, physiotherapists within the MBS pathway [[15], [16], [17], [18], [19]]. Consequently, their attitudes and experiences remain largely unexplored. Moreover, existing evidence indicates that lifestyle support for MBS patients is often suboptimal [20,21], highlighting the need to better understand care delivery from the perspective of LHCPs to inform improvements in patient care.

Given these gaps, this study explores the attitudes and experiences of LHCPs who care for MBS patients in the Netherlands. Specifically, we examine how LHCPs perceive MBS and its patients and the challenges they encounter when caring for this group. Insights from LHCPs’ perspective can inform HCP education and contribute to improved patient care. Moreover, because healthcare systems worldwide face similar challenges in managing obesity, these findings hold international relevance for strengthening multidisciplinary bariatric care and supporting long-term patient outcomes.

## 2 Methods

### 2.1 Design

A qualitative descriptive approach using semi-structured interviews was used [22,23]. This well-established exploratory methodology is widely used in healthcare research and is adaptable to diverse contexts, particularly for examining HCPs’ perspectives. It facilitates exploration of attitudes and experiences to identify challenges or gaps in practice and informs improvements in patient care.

### 2.2 Study setting

This study took place in the Netherlands, where MBS is provided within a multidisciplinary care pathway. LHCPs, such as dietitians, physiotherapists, and, in some centers, lifestyle or exercise coaches, are actively involved throughout the MBS care pathway.

### 2.3 Recruitment of participants

Participants were eligible if they were (1) registered LHCPs, such as dietitians, nutritionists, lifestyle coaches, physiotherapists, or other lifestyle-focused professionals, and (2) had experience caring for patients with obesity and MBS in the pre- or postoperative phase. Recruitment occurred via email (367 invitations), professional networks, social media (e.g., LinkedIn), and snowball sampling, yielding 38 responses. Two respondents were ineligible - one had no obesity experience, and one only worked with childhood obesity. Eight interested participants could not take part due to time constraints. The remaining 28 were contacted by the first author (FG) or a student assistant with study information and consent forms. Interviews were scheduled at participants’ convenience; four did not follow up, resulting in 24 completed interviews. During the interviews, two HCPs reported general obesity experience but no MBS-specific experience, leaving 22 participants for analysis. No additional interviews were conducted.

### 2.4 Data collection and procedure

Data were collected via semi-structured, one-on-one online interviews on Microsoft Teams between January and May 2025, averaging 43 min (range 30–65). The interview guide (Appendix A) ensured topic consistency while allowing participants to share new ideas and experiences. Interviews were conducted by the first author (FG) and a student assistant. Interviews began with general questions about participants’ professional backgrounds, followed by a section on attitudes toward and experiences with obesity, including perceptions of its causes, comfort discussing it with patients, challenges in practice, and views on effective interventions. The final section focused on attitudes toward experiences with MBS and patients undergoing the procedure, covering perceptions of its effectiveness, attitudes toward these patients, and challenges experienced in caring for this patient group. This study specifically analyzes findings from this final section.

### 2.5 Data analysis

Interviews were audio-recorded and transcribed verbatim. Transcripts were anonymized by removing names and identifying information, and each participant was assigned a unique study reference number. Data were analyzed in Atlas. ti version 24 [24] using thematic analysis with both deductive and inductive coding, allowing patterns in HCPs’ accounts of caring for MBS patients to be identified and interpreted.

The first author (FG) familiarized herself with the data through repeated transcript readings. An initial codebook was developed deductively from the interview guide, with additional inductive codes added to capture unanticipated insights. The research team collectively reviewed and refined the codebook to ensure coherence and consistency. All transcripts were systematically coded by the first author, and six transcripts were co-coded by the team to validate the findings. The coded data were then examined to identify emerging themes, connections among themes, and patterns across interviews. Through iterative reflection and discussion with the research team, themes were refined and organized as presented in the results, providing a comprehensive, contextually grounded account of HCPs’ perspectives and experiences. To ensure credibility, we shared the findings with participants; three responded and confirmed the results. Reporting followed the COREQ checklist (Appendix B) [25].

### 2.6 Ethics

Wageningen University Research Ethics Committee approved this study on December 19, 2024 (2024-252–A). Participation was voluntary, and participants were informed of their right to skip questions or discontinue the interview at any time. All participants provided oral consent before the interviews.

## 3 Results

Twenty-two participants were included: 13 dietitians, 8 lifestyle coaches, and 1 physiotherapist/manual therapist. Most (n = 18) worked in primary care and 4 worked in secondary care. All lifestyle coaches and the physiotherapist/manual therapist were employed in the two-year Combined Lifestyle Intervention (CLI) program [26], which promotes healthier weight through nutrition, physical activity, stress, and sleep management. Some participants held dual roles, such as dietitians who were also accredited as lifestyle coaches. See Table 1 for participant characteristics.

**Table 1:** Participant characteristics (N = 22).

Interview #	Profession	Level of Care	Age	Gender	Years of experience	Experience with MBS	Work Environment	Extra Notes
**P1**	Dietitian	Primary	41	Woman	15–16 years	Pre & post-surgery	Outside hospital	Teaches lifestyle coaches
**P2**	Dietitian	Primary	29	Woman	6 years	Post-surgery	Outside hospital/CLI^a^	Teaches lifestyle coaches
**P3**	Dietitian	Primary	29	Woman	6 years	Pre-surgery	Outside hospital	
**P4**	Dietitian	Primary	50+^b^	Woman	35 years	Pre & post-surgery	Outside hospital	
**P5**	Dietitian	Secondary	45	Woman	25 years	Pre & post-surgery	Hospital	Also accredited as a lifestyle coach
**P6**	Dietitian	Primary	34	Woman	10 years	Pre & post-surgery	Outside hospital	
**P7**	Dietitian	Primary	24	Woman	4 years	Pre & post-surgery	Hospital	
**P8**	Dietitian	Secondary	25	Woman	4 months	Pre & post-surgery	Hospital	
**P9**	Dietitian	Primary	53	Woman	3 years	Pre & post-surgery	Outside hospital	Also accredited as a lifestyle coach
**P10**	Dietitian	Primary	62	Woman	40 years	Pre & post-surgery	Outside hospital	
**P11**	Dietitian	Secondary	37	Woman	12 years	Pre & post-surgery	Hospital	
**P12**	Dietitian	Secondary	26	Woman	1 year	Pre- surgery	Hospital	
**P13**	Lifestyle coach	Primary	60	Man	7 years	Post-surgery	CLI	
**P14**	Lifestyle coach (ex-Dietitian)	Primary	40	Woman	2 years	Post-surgery	CLI	Also accredited as a Dietitian
**P15**	Lifestyle coach/Physiotherapist	Primary	29	Man	2.5 years (plus 8–9 years as physiotherapist)	Post-surgery	CLI	
**P16**	Physiotherapist/Manual Therapist	Primary	52	Woman	30 years	Post-surgery	CLI	
**P17**	Lifestyle coach	Primary	41	Woman	–	Post-surgery	CLI	
**P18**	Dietitian	Primary	39	Woman	18 years	Pre & post-surgery	Outside hospital	
**P19**	Lifestyle coach	Primary	53	Woman	3 years	Post-surgery	CLI	
**P20**	Lifestyle coach	Primary	52	Woman	6–7 years	Pre & post-surgery	CLI	
**P21**	Lifestyle coach	Primary	55	Woman	3 years	Post-surgery	CLI	
**P22**	Lifestyle coach	Primary	28	Woman	7–8 years	Post-surgery	CLI	

a **CLI:** Combined Lifestyle Intervention.

b Exact age participant P4, Dietitian unknown.

**Fig. 1:**
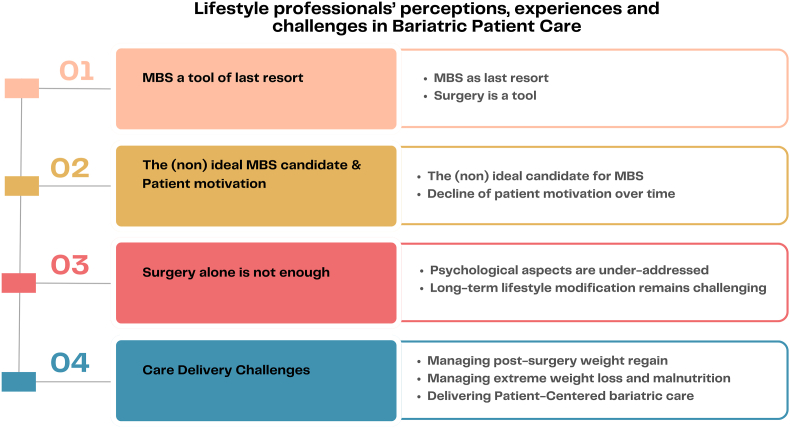
Thematic overview of themes and sub-themes. **Note**: MBS refers to metabolic and bariatric surgery.

Fig. 1 provides an overview of the four core themes and their subthemes: (1) *MBS as a tool of last resort*, (2) *The (non)ideal MBS candidate and patient motivation,* (3) *Surgery alone is not enough*, and (4) *Care delivery challenges*, along with their associated subthemes. Table 2, Table 3, Table 4, Table 5 provide representative participant quotations for each subtheme and are cited under the corresponding theme headings in the results section.

**Table 2:** Illustrative Quotes for Theme 1: MBS a tool of last resort.

Core themes	Sub-themes	Examples of supporting quotes
**1. MBS a tool of last resort**	**1.1 MBS as last resort**	“I always find it a very intense choice, because, well, you are letting someone cut into your body. And I also think it's kind of a very desperate choice. […] I do understand why someone would do it. If you really want it and you have certain conditions or diseases that get worse because of your excess weight, then it's a kind of last resort.” (**P2, Dietitian**)
		“Of course, it's quite a major procedure. For me it wouldn't be a first option.” (**P9, Dietitian**)
		“For us it is really the final station.” (**P17, Lifestyle coach**)
		“I do see it somewhat as a kind of last resort. But I also really understand people very well if they've tried everything and nothing works. And they have diabetes and knee problems. And the body just won't cooperate. Then I understand it very well.” **(P4, Dietitian**)
		“It really is a last resort. I truly see it as something you do when nothing else works. Or when the situation is really urgent, when someone has things like diabetes or cardiovascular disease. It's a bit like choosing between two evils, that's how I see it.” (**P6, Dietitian**)
		“For us it's actually the final station. Intervening in an intact body. So first we look at: can we do something with lifestyle? If not, can we do something with medication? If not, is bariatric surgery a good solution … ?” (**P16, Physiotherapist**)
		“I don't know if I would personally recommend it to patients when they have overweight. I really see it as a last resort, which is also what those patients say themselves.” **(P8, Dietitian**)
	**1.2. Surgery is a tool**	“A gastric reduction is a great tool, not a miracle cure. It helps you get started. It makes you feel less hungry, but you still have to do the rest yourself.” (**P7, Dietitian**)
		“I do not think that a gastric reduction is going to solve everything. […]. For example, poor eating habits …” (**P8, Dietitian**)
		“Sometimes it can just help people. And it can give them that little push.” (**P9, Dietitian)**
		“It is a tool for obesity. But behaviour and eating patterns also need to be adjusted. It is not a miracle cure.” (**P10, Dietitian**)
		“A gastric reduction can really give you a push in the right direction, but you still have to put in the work.” (**P14, Lifestyle coach**)
		“I assume that everyone is told that they indeed have to adjust their lifestyle and that this is a tool to help achieve that, but that it is still hard work.” (**P15, Lifestyle coach**)
		“It is certainly not a miracle cure [ …] People need to know in advance what it takes to live healthily. … Bariatric surgery is only a catalyst for a healthy lifestyle.” (**P16, Physiotherapist**)
		“He called it a kind of emergency button that you can press once, like a metabolic reset. That already gave me a much clearer picture: Oh, this does much more in your body than just a gastric reduction …” (**P17, Lifestyle coach**)

**Note:** MBS refers to metabolic and bariatric surgery.

**Table 3:** Illustrative Quotes for Theme 3: Patient motivation and engagement.

Core themes	Sub-themes	Examples of supporting quotes
**2. The(non) ideal MBS candidate and patient motivation**	**2.1 The (non) ideal MBS candidate**	“It is certainly effective for a specific target group. And it's also nice that it's there. For another target group, I think, ‘perhaps a little more could have been done beforehand’.” (**P1, Dietitian**)
		“I think, especially for people who are really motivated and have already made a lifestyle change but just can't quite manage that last bit, that it's a really wonderful thing.” (**P22, Lifestyle coach**)
		“Some patients could really benefit from it, it helps them a lot, but there are also many patients for whom the mindset issue is so much bigger and requires so much more attention than gastric bypass surgery can provide.” (**P8, Dietitian**)
		“I saw quite a few people in the pre-operative phase. And some of them really do their best. They adjust everything. And you can just see that they are so motivated. They really want to go ahead with the operation. But with others, you have your doubts. […] These people may have thought of the operation as a miracle cure. And of course, that can be really disappointing afterward. And that bit of a disappointment is something they have to deal with.” (**P1, Dietitian**)
		“That is the biggest challenge: to change those thoughts or that mindset” (**P14, Lifestyle coach**)
		“I sometimes still feel that people are a bit too casual about it. It's just a relatively easy and quick solution to their problem.” (**P22, Lifestyle coach**)
		“I have seen people who have taken it far too casually and wanted it at all costs […] There are many psychological factors involved - emotional eating gets in the way. Which can then lead to disappointment” (**P6, Dietitian**)
	**2.3 Decline of motivation over time**	“In the beginning, motivation is usually good, but after a year or two, it always declines a bit.” (**P6, Dietitian**)
		“You have to keep working on that behaviour change yourself. So that remains very difficult, and in that process the motivation sometimes drops a bit as well.” (**P11, Dietitian**)
		“I think they are really super motivated in that moment – [the beginning] […] The relapse [in motivation] usually only comes after two years or so …” (**P8, Dietitian**)
		“In terms of motivation, if someone has already had a gastric reduction and still hasn't managed to maintain their weight […] then I think: Okay, I really need to be extra alert to their long-term motivation.” (**P22, Lifestyle coach).**

**Note**: MBS refers to metabolic and bariatric surgery.

**Table 4:** Illustrative Quotes for Theme 2: Surgery alone is not enough.

Core themes	Sub-themes	Examples of supporting quotes
**3. Surgery alone is not enough**	**3.1 Psychological aspects are under-addressed**	“If someone fills out a food diary prior to screening, showing that they eat four or three times a day and only eat chicken and fries in the evening, then I also think: How are we going to make so many changes when we proceed with the surgery? There is so much that needs to be adjusted, and that simply doesn't always work. […] If it were up to me, I would see patients much more often, so I could better screen whether or not they would be eligible, looking at how much they would have to change their diet.” (**P8, Dietitian**)
		“I once had a man [patient], a nurse working in addiction care, who called himself addicted to food. I found that really remarkable. And he ended up having gastric bypass surgery. Which made me think: How did you even get through the screening process?” (**P4, Dietitian**)
		“They often give advice such as, “Well, maybe it would be useful to talk to a practice nurse or psychologist about certain topics.” But that's as far as it goes, and it would be nice if more attention were paid to this.” (**P7, Dietitian)**
		“I feel that the [psychological] pre-operative phase is somewhat lacking. Why do I say that? Because people come back to me after the operation having gained weight again - precisely because they are still struggling with emotional eating. That is the group I see in particular.” (**P1, Dietitian**)
		“We have noticed that [psychological] support has also decreased somewhat during and after the corona pandemic” **(P4, Dietitian)**
		“What I often see is that too little attention is paid to the mental aspect of the process. […] It would be nice if more focus were placed on that, because I also think that some gastric bypasses might not even be necessary then.” (**P7, Dietitian**)
	**3.2 Long-term lifestyle modification remains challenging**	“She came to me for the [CLI] program because she had simply gained weight again, a few years after her gastric bypass surgery. Because nothing had fundamentally changed. After the surgery she lost a lot of weight, but she hadn't made any changes to her lifestyle**.”** (**P19, Lifestyle coach)**
		“I hear back that the CLI really complements the support they receive from, for example, the [obesity clinic], or wherever else they have sought help. Because, in their eyes, that [support from the clinic] isn't always sufficient.” (**P17, Lifestyle coach)**
		“Bariatric surgery is a huge procedure. And if those [people] end up back in a CLI, then we haven't done something right somewhere. I think medical errors—if you perform such a major procedure and those people become obese again, then you haven't paid enough attention at the outset[…] Either the pre-operative process wasn't done properly, or the aftercare wasn't good enough.” (**P16, Physiotherapist**)
		“Basically, obesity is a behavioral problem. People have to cope in a very challenging Western world. And they haven't managed to do that. […] And then we just go ahead with the gastric reduction. I am really in favor of it, but not before we have talked with these people about behavior and behavior change. And I'm not just talking about what to eat, but also about the situations they are in […] That they need to move more, and that being outdoors and getting enough sleep are important for them. So little attention is paid to this, and so little guidance is provided. I have my doubts about the long-term effectiveness of bariatric surgery if that [pre-operative process] isn't done very well.” (**P16, Physiotherapist**
		“You can say, ‘willpower, willpower,’ but at some point, that runs out. And if you've lived your whole life like that, it really is a tough struggle.” (**P13, Lifestyle coach**)

**Table 5:** Illustrative quotes for theme 4: Care delivery challenges.

Core themes	Sub-themes	Examples of supporting quotes
**4. Care Delivery Challenges**	**4.1 Managing Post-Surgery Weight Regain**	“Five or six years after the surgery, I see them again. And unfortunately, with almost all of these people, we see the weight starting to increase again. So that's difficult. I see them regularly.” (**P10, Dietitian**)
	**4.2 Managing extreme weight loss and malnutrition**	“The risk of osteoporosis is, of course, already higher … You don't want to make all those kinds of things worse. And muscle breakdown, and so on. So, it really becomes a completely different kind of trajectory.” (**P4, Dietitian**)
		“Occasionally, there is who someone loses too much weight and can't seem to get a grip on it, because their stomach is much smaller, and they can't eat enough. For these people, it's very challenging, because we can't just say, ‘Eat a bit more and you'll gain weight. ‘That doesn't work. They need extra attention. And sometimes we need to tube feeding, otherwise they won't gain weight or be able to control their weight. That's the other extreme. It's not very common, but these are often the more challenging clients, where you need to figure out how to get their weight under control.” (**P7, Dietitian).**
		“What I also found difficult is that after the surgery there were often patients who still developed complaints, for example dumping symptoms, or who became severely undernourished because they could hardly keep anything down and were vomiting frequently. That is really quite tough, also as a dietitian, because there is actually very little literature available on what you can do about this.” (**P12, Dietitian**)
	**4.3 Delivering Patient-Centered bariatric care**	“You just follow the guidelines with eating six times a day, not eating and drinking at the same time, high-protein … So basically, you try to follow that [guideline] as closely as possible. Then you're really working with knowledge [transmission].” **(P1, Dietitian**)
		“I've had a few who were operated on and still wanted to lose a few more kilos. But for those people, I can do very little, because they are following a very specific schedule, and there's very little room for deviation.” (**P15, Lifestyle coach**)
		“That [bariatric] program is very structured. They really must do certain things. Whereas in other conversations with people, you might want to explore: What suits you? What goals do you want to achieve? Here [in this program] you are quite directive. So, you say: ‘Well, it has to be done like this.’ And of course you can do this in different ways, but certain basic rules are the same for everyone. And sometimes, yes, I do feel quite strict about it.” (**P18, Dietitian**)

### 3.1 Theme 1: MBS a tool of last resort

LHCPs viewed MBS as a last-resort intervention after other approaches failed. This theme, including the subthemes *MBS as Last Resort* and *Surgery as a Tool*, reflects LHCPs’ attitudes toward MBS and their patients. Table 2 presents illustrative quotes.

#### 3.1.1 MBS as last resort

Almost all LHCPs (n = 20) described MBS as a “last resort”, reserved for individuals who have *truly* tried all other weight-loss strategies without success or whose weight poses greater health risks than the surgery itself. MBS was seen as invasive, irreversible, and riskier than less intensive treatments like medication. Consequently, LHCPs recommended it only after other options failed, viewing surgery as a final, necessary step in obesity management.

LHCPs note that many patients also view surgery as a last resort. LHCPs describe that these patients often feel emotionally drained and powerless due to their lifelong battles with obesity and repeated unsuccessful weight loss attempts. For them, surgery represents a final opportunity for better health and quality of life. LHCPs stress that the decision to undergo MBS is not taken lightly, as reflected in the quote: *“Patients don't do it on a whim; it's not for fun*” (**P4, Dietitian**). Even with the risks involved, LHCPs express understanding and support for their patients' choices. One dietitian explained their supportive approach: “*When someone says, ‘I want to have the surgery,’ I try to stay positive: ‘Well done for making that choice. This could actually be a good solution for you*’” (**P9**, **Dietitian**).

#### 3.1.2 Surgery is a tool

All LHCPs described MBS as an effective medical intervention. They viewed it as a supportive tool that helps facilitate weight loss, while rejecting the idea of it being a miracle cure. From their perspective, surgery provides a physiological advantage and a metabolic reset that reduces hunger, acting as a catalyst for adopting healthier lifestyle behaviors. However, LHCPs emphasize that the success of MBS relies on the active engagement of patients with behavioral factors (e.g., diet and exercise) and psychological factors (e.g., emotional eating) that contribute to obesity, and that this requires substantial effort and commitment from patients both before and after surgery. These beliefs reinforce the view of MBS as a supportive tool rather than a passive solution to obesity. LHCPs also acknowledged that the process is far from easy due to potential postoperative side effects, such as dumping syndrome, vitamin deficiencies, malnutrition, and the risk of addiction transfer (e.g., alcohol or energy drinks), which patients must learn to manage.

### 3.2 Theme 2: The (non) ideal MBS candidate and patient motivation

This theme reflects LHCPs' views on the ideal MBS candidate, distinguishing ‘ideal’ from ‘non-ideal’ characteristics, and highlights patient challenges experienced by LHCPs. Table 3 presents illustrative quotes for each subtheme.

#### 3.2.1 The (non) ideal candidate for MBS

LHCPs differentiate between two types of patients seeking MBS: those who exhibit the qualities of an “ideal candidate” for MBS and those who are hesitant to change or view surgery as a “quick fix”, making them a less ideal candidate. This distinction, according to LHCPs, separates MBS patients who are more likely to have successful outcomes after surgery from those less likely to achieve success. LHCPs described the ideal candidate as someone who has *truly* exhausted all other options to lose weight but continues to face barriers beyond their control. Ideal patients were also described as extremely motivated, individuals who actively work to change their behaviors and fully commit to the lifestyle required after MBS. These individuals were perceived as someone who would do everything within their control to support a successful surgical outcome.

Non-ideal candidates were described as individuals who are resistant to change, for example, those unwilling to adjust their mindset around physical activity and eating practices or who struggle to follow advice. LHCPs viewed these patients as unsuitable because they had not yet developed the lifestyle changes required for MBS and, due to their unwillingness to adjust, might even find ‘tricks’ to bypass dietary recommendations after surgery. According to LHCPs, such patients are more likely to see surgery as a ‘quick fix’ or miracle cure, perceiving it as an easy solution requiring little effort. One major challenge is addressing this mindset before surgery, which can be difficult to change. As one dietician reflected: “*There are also many patients for whom the mindset issue is so much bigger and requires so much more attention than gastric bypass surgery can provide.*” (**P8, Dietitian**). This often leads to disappointment when patients realize that surgery is not an endpoint but the start of a lifelong commitment to healthier habits.

#### 3.2.2 Decline of patient motivation over time

LHCPs characterize most preoperative patients as highly motivated, noting that they work hard to meet prescreening expectations, such as weight-loss targets and lifestyle changes. LHCPs emphasized that this motivation is not only practical but also deeply personal, driven by patients' belief that this is their ‘last chance’ to lose weight and regain control of their health.

After surgery, this motivation is often reinforced by fast and visible weight loss. However, many LHCPs express skepticism about the durability of this initial motivation, observing that it frequently declines after the first or second year, once the immediate benefits, such as rapid weight loss, lessen and everyday challenges resurface. It is in this later stage that LHCPs believe genuine, sustained behavioral change becomes essential. They described how patients often struggle to maintain new habits and may revert to long-standing maladaptive coping behaviors, especially during periods of emotional stress or loss. At the same time, LHCPs noted that this pattern is not unique to MBS patients; they have observed similar difficulties among individuals who lose weight through other interventions, confirming that maintaining long-term weight loss is a broader challenge rather than one specific to MBS.

### 3.3 Theme 3: Surgery alone is not enough

All LHCPs stressed that, in their view, the root causes of obesity are psychological and behavioral, issues that surgery alone cannot resolve. One LHCP illustrated this by pointing first to the stomach and then to the head, “*Cutting? Here [pointing to the stomach]. But the problem is here [pointing to the head].*” (**P14, Lifestyle coach)**, while another stated, “*You're not undergoing brain surgery … So lifestyle changes are also necessary*.” (**P13, Lifestyle coach**). These comments underscore the limits of MBS and its reliance on patients' psychological and behavioral changes for long-term success. Over half of the HCPs (N = 13) felt that these critical aspects are inadequately addressed in the surgical pathway. Quotes for Theme 3 are presented in Table 4.

#### 3.3.1 Psychological aspects are under-addressed

**L**HCPs emphasized that the psychological aspects of obesity are often overlooked or under-addressed in MBS care. They stressed the need for comprehensive psychological assessments *before* surgery, believing this could lead to more sustainable outcomes or, in some cases, even eliminate the need for surgery.

Several LHCPs (n = 7) noted gaps in the screening of psychological challenges at the beginning of the MBS trajectory. While pre-operative screenings usually involve a psychologist, dietitian, and specialized nurse, some providers questioned whether this approach effectively identifies deeper issues such as emotional eating, addiction, or low patient motivation. One LHCP emphasized that counseling is often only suggested rather than prioritized, recommending a greater focus on this area, especially for patients with behavioral issues like food addiction or complex histories. Binge eating and emotional eating were highlighted by numerous providers as core unresolved issues that persist long after surgery. These patients may benefit from seeing a psychologist or behavioral therapist first.

When these psychological aspects are inadequately addressed, patients may internalize feelings of failure, blaming themselves for difficulties in losing or maintaining weight, as one physiotherapist remarked, “*It's never the patient's fault, it's always ours. We simply didn't give them enough information. […] Later, when those people relapse, they say, ‘Yeah, it's also my fault, because I got plenty of* support*, but I still managed to mess it all up’*.” (**P16, Physiotherapist).** The consensus among LHCPs is that while the operation may reduce hunger signals, it does not resolve the underlying behavioral triggers that lead individuals to eat in response to stress, sadness, or other emotions.

#### 3.3.2 Long-term lifestyle modification remains challenging

All LHCPs agreed that sustained lifestyle and behavioral change are essential for long-term success following MBS. Pre-operative preparation was deemed necessary to ensure patients clearly understand the long-term lifestyle requirements related to, for instance, nutrition, physical activity, and stress management. However, participants highlighted persistent challenges in the structure of care aimed at lifestyle change. While patients receive information and support before and after MBS, LHCPs emphasized that the depth and duration of this support are often insufficient for long-term change. Most HCPs (N = 17) reported that patients returned to lifestyle programs several years post-surgery due to weight regain, a phenomenon that was particularly noticeable among LHCPs employed in the CLI programs.

Maintaining initial weight loss requires what two LHCPs call “military discipline*”*, a level of self-control that can be difficult even for those with a healthy BMI. As one dietitian explained, “*The first year is easy - well, easier - but after that, it doesn't happen automatically, and you really have to maintain it [****the weight loss]****. That sometimes requires a kind of military discipline, and that's very difficult*.” (**P2, Dietitian**).

LHCPs recognize that sustaining weight loss involves ongoing self-management, not just initial effort. Adhering to dietary and lifestyle changes can be tough for anyone, not just those actively trying to lose weight. Therefore, they stress the need for lifelong follow-up beyond medical monitoring to effectively support the maintenance of lifestyle changes and weight loss post-surgery.

### 3.4 Theme 4: Care delivery Challenges

This theme highlights the challenges LHCPs face in caring for MBS patients, from managing weight regain or excessive weight loss to delivering patient-centered care. Table 5 presents illustrative quotes for each subtheme.

#### 3.4.1 Managing post-surgery weight regain

Most LHCPs (N = 17) who treated patients post-surgery encountered them primarily due to weight regain. Several HCPs described significant challenges in supporting patients who experienced weight regain after MBS. Once surgery, the ‘final option’, had been used, both patients and providers often struggled to identify alternative ways to manage obesity. As one dietitian reflected: “*How can someone lose weight again if they want to? After all, they've already had surgery, right? That was already one option. What else can they do?*” (**P1, Dietitian**) This raised concerns about the limited effective interventions available when surgery is unsuccessful.

LHCPs also struggled to maintain patient motivation when individuals could not sustain weight loss after MBS. While some LHCPs reverted to lifestyle programs or weight-loss medications, they expressed doubts about their effectiveness post-surgery. One lifestyle coach remarked, “*I have a woman who has really tried everything, including bariatric surgery. She is now allowed to use medication to lose weight, but only after participating in the CLI. That's one of those situations where I think I probably can't do much for her, except be there for her during the activities I organize. […] Will the CLI really make much difference to her weight? I'm afraid not …*” (**P21, Lifestyle coach**).

Overall, these LHCPs experienced uncertainty and frustration when managing weight regain, feeling constrained by limited expertise and skills and treatment options. Their reflections highlight both the emotional and professional challenges of supporting patients for whom BS did not produce lasting success.

#### 3.4.2 Managing extreme weight loss and malnutrition

While MBS is intended to treat obesity, several LHCPs (N = 6), all dietitians, explained that some patients experience excessive weight loss postoperatively, leading to extreme underweight and malnutrition. One LHCP described a patient who weighed 39 kg after surgery: “*We also see people who lose too much weight and then develop eating disorders. […] They often come to us after the operation. I have a lady who now weighs only 39 kg. That was never the intended outcome, of course*.” (**P4, Dietitian**).

LHCPs explained that in these cases, the challenge shifts from treating obesity to managing malnutrition and underweight. This involves preventing patients from dropping below a healthy weight and minimizing the risk of complications such as osteoporosis, muscle wasting, and general malnutrition. Providers also explain that the small stomach size after surgery limits the ability to eat more, making recovery and weight stabilization difficult. As one dietician pointed out, simply telling patients to “eat a bit more and you'll gain weight” is ineffective (**P7, dietitian**). For patients who dropped too far into the underweight range and could not regain weight, tube feeding was sometimes required to stabilize nutritional status. However, one dietician noted that surgeons often preferred not to initiate tube feeding, arguing that patients who received it frequently did not lose weight afterward. Another LHCP expressed frustration that they do not have enough time to care for these complex cases. From their perspective, standard insurance-covered consultation times (3 h per year) were structurally insufficient to manage the complex challenges of malnutrition. Postoperative complications, such as dumping syndrome or severe undernutrition with frequent vomiting, further complicated patient care. LHCPs described managing these patients as ‘quite tough’ and noted that the limited availability of evidence-based guidelines on how to manage these cases made it even more challenging to provide adequate and effective support.

#### 3.4.3 Delivering patient-centered bariatric care

LHCPs, particularly those involved before and immediately after surgery, such as dietitians, often felt constrained in the care they could provide to patients undergoing MBS. Dietitians explained that the goals for MBS are primarily focused on weight loss and are typically established before the surgery, often by the surgeon.

While managing expectations can help ensure that these goals remain realistic, LHCPs explained that their work is frequently dictated more by the predefined MBS trajectory than by the individual needs of the patient. Much of their interaction with patients focused on following dietary guidelines specific to the MBS pathway, leaving little room to explore personal experiences, underlying causes of obesity, or adapt treatment plans to individual needs, as one dietitian explained: “*In the obesity clinics, It's generally more like:* *“Here's a nutrition plan or a food diary. Just follow that … And you need to lose 5 kilos before surgery.’ It's difficult to make the process more personalized*.” (**P8, Dietitian**). Consequently, care was often directive and information-based, with dietitians describing their role as primarily conveying knowledge rather than engaging in shared decision-making or providing individualized support. This lack of flexibility was also felt when supporting patients after surgery.

## 4 Discussion

This study explored the attitudes and experiences of LHCPs providing care to MBS patients. To date, it is the first study to specifically examine their experiences. The study's findings highlight that although LHCPs generally expressed positive attitudes toward MBS and its effectiveness, this optimism was tempered by recognition that surgery alone is insufficient. They emphasized that long-term outcomes depend on addressing lifestyle and psychological factors, and many described challenges in providing this support, particularly in post-surgical primary care.

MBS is a tool, not a miracle cure, and still requires considerable patient effort before and after the procedure; it is therefore best considered a last resort when other weight-management options have failed for reasons beyond the individual's control. This view was prominent among LHCPs in our study and aligns with previous research among primary care practitioners [[27], [28], [29], [30]]**.** Viewing MBS as a last resort stemmed from the idea that surgery, although perceived as the most effective treatment for obesity, was considered invasive, risky, and irreversible compared to, for example, medication. Consequently, it would be recommended as a last option.

LHCPs consistently describe the ideal candidate for MBS as someone who is highly motivated and committed to making the necessary lifestyle changes before and after the procedure. These qualities, often referred to in the literature as readiness for behavioral change [31,32], are traditionally considered important predictors of surgical success. However, LHCPs note that although most individuals begin their surgical journey highly motivated, sustaining this motivation is challenging. Many patients experience a significant decline in motivation after about two years, as they struggle to maintain lifestyle changes due to emotional distress, ongoing stressors, and reversion to previous habits - a phenomenon supported by patient experiences [33]. Additionally, LHCPs point out that the expectations for strict adherence to lifestyle modifications are often unrealistic, arguing that demanding “military-level discipline” is difficult, even for individuals without obesity. These fluctuations in motivation, combined with unrealistic expectations, create significant challenges for patients and LHCPs. While readiness and motivation are important, LHCPs emphasize the need for long-term lifestyle care to sustain positive patient outcomes.

In addition to behavioral and lifestyle support, HCPs emphasized the importance of addressing psychological factors to effectively support MBS patients, an area that remains inadequately addressed in current bariatric pathways, corroborating findings from non-LHCPs [27,34]. Surgery alone was seen as insufficient to resolve underlying psychological contributors to weight gain, such as emotional eating. Pre-operative screening was considered an important initial step; however, many HCPs questioned whether existing assessments effectively identify patients with significant psychological challenges, which could compromise patient outcomes if untreated. This concern aligns with recent research showing that screening and treatment for disordered eating in MBS patients remain inconsistent and lack a clear evidence base [35].

The findings suggest that, from the LHCPs' perspectives, lifestyle and psychological factors influencing patient outcomes are not always sufficiently addressed. This may, in turn, contribute to post-operative maladaptive behaviors and weight regain. HCPs indicated that enhancing pre-surgical screening and integrating behavioral and psychological support both before and after surgery could improve patients’ ability to sustain lifestyle changes - recommendations supported by surgical and behavioral literature [3,9].

LHCPs also described challenges in meeting the support and care needs of people after MBS. In particular, they noted difficulties in caring for patients experiencing weight regain, as well as those presenting with excessive weight loss or malnutrition. In cases of weight regain, both patients and providers often struggle to identify remaining treatment options; with lifestyle interventions and MBS already attempted, there is a sense that all avenues have been exhausted, leading to the reintroduction of lifestyle strategies that were previously unsuccessful. Challenges related to excessive weight loss and malnutrition were described more frequently by dietitians outside of hospitals (primary care), who highlighted resource constraints, limited evidence-based guidance, and insufficient insurance-covered consultation time - factors that create time pressures and limit their ability to provide comprehensive care. Surprisingly, patients seek support from LHCPs in primary care despite five years of hospital-based follow-up, suggesting post-surgery support in secondary care may be insufficient. It is also important to mention that these care-delivery challenges are not unique to dietitians; they also mirror those reported by general practitioners supporting post-bariatric patients in primary care [27,36] and highlight the need for training among these HCPs to better support patients.

When caring for MBS patients, dietitians have reported challenges in balancing protocol-driven requirements with tailored care that considers individual circumstances. They reported frequently using directive communication, such as instructing patients to eat six times a day to encourage adherence, even when such recommendations may not align with the patients' preferences or lived experiences. Unintentionally, this type of communication may inadvertently damage the patient-provider relationship [37] and reinforce weight stigma [38]. However, cultural context also shapes how patients perceive this style of guidance. In many non-Western settings, prescriptive advice is valued and associated with professional expertise [37,39]. These findings suggest that while standardized protocols promote safety and consistency [40], they can limit focusing on individual experiences and underlying factors contributing to obesity [34]. The experiences described highlight the complexity of the MBS trajectory and the need for individualized care pathways. Effective post-operative care requires balancing such protocols with flexible, patient-centered approaches that respect personal needs and avoid stigma.

### 4.1 Strengths and limitations

To our knowledge, this is the first study to explore the perspectives of LHCPs involved in bariatric care. Our study goes beyond describing the practical roles of LHCPs, such as expected responsibilities and routine clinical activities, which has been the focus of most previous research [[15], [16], [17], [18], [19]]. Instead, it explores their unique experiences and challenges in caring for MBS patients. The study highlights not only how LHCPs perceive and support these patients, but also what they identify as necessary to improve care. For example, LHCPs reported lacking sufficient knowledge, skills, and evidence-based guidelines to address the specific needs of MBS patients, highlighting the need for targeted education and resources to enable them to provide better care. Moreover, their experiences provide valuable insights into how lifestyle and behavioral expertise can complement surgical treatment and highlight the need for stronger interdisciplinary integration.

One limitation is that participants often worked with patients struggling post-surgery, as those doing well tend to discontinue follow-up, potentially creating a negative bias in perceived outcomes. Additionally, the perspectives of certain LHCPs involved in MBS care, such as exercise physiologists, occupational therapists, physical therapists, and health coaches, were not included, so the findings reflect only a limited range of experiences in MBS care. Furthermore, all participants practiced within the Dutch healthcare system, which may limit the transferability of our findings to other countries. Nevertheless, MBS care in countries such as the United Kingdom, the United States, Canada, Australia, and several European nations shows common organizational features [[41], [42], [43]], including structured pre- and post-surgery pathways, defined follow-up periods, and multidisciplinary teams integrating LHCPs. Patients in these contexts also present challenges similar to those observed in this study [27,34]. These parallels in care organization and patient experiences indicate that the findings may be transferable across other Western healthcare settings.

While data saturation was not sought, the interview provided rich, meaningful, and recurring insights. Consistent with qualitative research practice, where saturation is understood as achieving sufficient data richness rather than a fixed number of interviews [44], additional interviews were judged unlikely to generate new insights for this underexplored professional group.

### 4.2 Future Research and practical Recommendations

Although this study focused on LHCPs working with MBS patients, most participants were employed outside hospital settings. The work context (primary vs. secondary care) appeared to shape experiences significantly. Dietitians working in community settings more often encountered patients with post-operative complications or suboptimal outcomes compared to their hospital-based colleagues, influencing their perspectives. All other non-dietitian LHCPs in this study worked outside hospital settings, so we were unable to determine whether their experiences also differed across settings. Moreover, the perspectives of lifestyle coaches and physiotherapists embedded within surgical teams were not represented. Future research should include these professionals to better understand how work settings influence perceptions of surgery and patient care. Such insights could inform guidelines for LHCPs in community settings to improve post-surgical support. Furthermore, future research should include the perspectives of a broader range of LHCPs to capture a more comprehensive understanding of their experiences in MBS care.

Overall, hospitals should consider integrating LHCPs, such as physiotherapists and lifestyle coaches, more formally into bariatric pathways to ensure continuity of care. Longitudinal and mixed-methods studies could further evaluate the impact of ongoing behavioral and psychological support on long-term weight maintenance, nutritional status, and quality of life.

## 5 Conclusions

Overall, the findings of this study suggest that while MBS is an effective treatment, it should be considered a starting point and facilitator, rather than a stand-alone or final intervention. Long-term outcomes depend on integrating psychological and behavioral care alongside surgery. LHCPs emphasized the importance of addressing the psychological aspects of obesity before surgery and strengthening lifestyle support both before and after the procedure to help patients build the self-management and behavioral skills needed for sustained success and better long-term outcomes.

Even before hospital-based follow-up ends and patients seek care in primary care, this study shows that - not only GPs [27,36] but also LHCPs - become main points of contact for ongoing support. However, LHCPs require additional training and time to provide adequate post-surgical care for MBS patients in primary care settings.

**Key take-home messages**

• LHCPs view MBS as an effective intervention but emphasize that long-term success fundamentally depends on lifelong behavioral changes and ongoing psychological support, rather than surgery alone.
• Managing post-surgical challenges - such as weight regain, excessive weight loss, and malnutrition - is difficult in routine practice, as specialized skills required or access to informational resources are often limited among LHCPs.
• As patients transition back to primary care, LHCPs often become key post-surgical points of contact alongside GPs, yet limited training, time, and strict care protocols constrain their ability to provide optimal long-term care.

## Ethical considerations

Ethical approval was obtained from the Wageningen University Research Ethics Committee on December 19, 2024 (2024-252–A). Participation in the study was voluntary. Informed oral consent was obtained from all participants, who were fully informed of their rights, including the option to withdraw from the study at any time. We maintained participant confidentiality by removing all identifying information from the data collected.

## Author contributions

Franshelis **K. Garcia:** Writing – review & editing, Writing – original draft, Visualization, Validation, Project administration, Methodology, Investigation, Formal analysis, Data curation, Conceptualization. **Bob C. Mulder:** Writing – review & editing, Validation, Supervision, Formal analysis, Conceptualization. **Eric J. Hazebroek:** Writing – review & editing, Validation, Supervision, Conceptualization. **Annemarie M.A.E. Wagemakers:** Writing – review & editing, Validation, Supervision, Formal analysis, Conceptualization. **Kirsten T. Verkooijen:** Writing – review & editing, Supervision, Formal analysis, Conceptualization.

## Declaration of artificial intelligence (AI) and AI-assisted technologies

Artificial intelligence (Grammarly, 2025) was used solely to assist with grammar correction during the preparation of this manuscript. The authors reviewed and edited the content as needed and take full responsibility for the content of the publication. No other artificial intelligence tools were used for data analysis, interpretation, or content generation.

## Funding

This research was supported by a grant from the Wageningen school of Social Sciences (WASS). Beyond payment to the research staff by 10.13039/501100004890Wageningen University & Research, this research did not receive any specific grant from funding agencies in the public, commercial, or not-for-profit sectors.

## Declaration of competing interest

The authors declare that they have no known competing financial interests or personal relationships that could have appeared to influence the work reported in this paper.

## Data Availability

The data generated and analyzed during the study are available from the corresponding author on reasonable request. Interview transcription data is not available to protect the confidentiality of the participants.
