# Supplementary material for: Attitudes and experiences of lifestyle healthcare professionals in the care of metabolic and bariatric surgery patients in the Netherlands
Source: Obes Pillars. 2026 Mar 25;18:100261. doi: 10.1016/j.obpill.2026.100261 (PMC13054046; doi:10.1016/j.obpill.2026.100261)
Supplement: Multimedia component 1 [file mmc1.docx]

**Appendix A: Interview protocol (Translated from Dutch version)**

**Before interview starts**

1. Introduce myself.
2. Thank the participant for their participation.
3. Explain the study.
4. Discuss informed consent:
   - Explain the points in the informed consent form regarding participation and careful handling of data.
   - Thank the participant for providing written consent and ask for verbal consent for video and/or audio recordings.
5. START RECORDING
6. Reassure the participant:
   - Emphasize that their perspective and experience are valuable.
   - Explain that there are no wrong answers.
7. Check if the participant has any questions.

**Section 1: Background questions:**

- Can you tell me about your background and how you became a ________ [**profession**]?
- How long have you been working as a ________ [**profession**]?
- Which aspects of your work give you energy or make you proud?
- What is your area of expertise as a ________ [**profession**]?
  - *Prompt:* Are there any specific topics you are particularly passionate about?

**Section 2: Experiences with (supporting people with) obesity**

- Can you tell me about your experiences supporting people with obesity?
- About how many patients with obesity do you see each week?
- What do you find to be the biggest challenges when supporting people with obesity?
- Can you share a moment when you felt particularly proud of your work with a patient who successfully managed their obesity?
- In your view, what are the main causes of obesity, and why?
- How do you determine whether an intervention or treatment has been successful for your patients with obesity?
- How do you usually feel when discussing obesity with your patients? Is it something you find easy to talk about? Why or why not?
  - ***Prompt:*** Are there specific terms or words you prefer to use when discussing obesity? Why?
- Which experiences have shaped the way you support or guide your patients?
- Do you feel your education has adequately prepared you to support or treat people with obesity?
  - If yes, which aspects of your education were particularly helpful?
  - If no, have you taken steps to supplement this, such as attending specialized training or workshops?
- In your view, what would an “ideal treatment for obesity” look like
  - ***Promp****t:* Who would be part of your team to effectively prevent and/or treat obesity?
- What do you think is needed to achieve this ideal approach to obesity treatment?
- What challenges or obstacles do you foresee in making this ideal a reality?

**Section 3: Metabolic Bariatric Surgery (MBS)**

Views on surgery and patients undergoing BS

- What are your general views on bariatric surgery as a treatment for obesity?
  - ***Prompt:*** Do you think it is an effective way to treat obesity?
- Have you ever referred someone for bariatric surgery?
- What are your general views about patients who undergo bariatric surgery?

Experience with MBS patients

- Have you worked with patients who have undergone, or are planning to undergo, bariatric surgery?
- Can you share your experiences with patients who have had bariatric surgery?
  - *Prompt:* What challenges or successes have you encountered in supporting them?
- How does supporting patients who have had bariatric surgery differ from supporting patients who have not undergone surgery?
- Do your expectations or treatment goals differ for patients who have had bariatric surgery compared with other patients with obesity?

**Section 4: Post-operative MBS support**

Approaches and expectations for BS patients

- How do you provide nutritional or lifestyle support to patients who have undergone bariatric surgery?
- In what ways does this approach differ from how you support other patients, if at all?
- How motivated do you think patients are to make lifestyle changes after bariatric surgery? Do you think they are able to maintain these changes in the long term?

***Prompt:*** What factors do you think influence their motivation?

Comparison with other treatments

- How do you compare bariatric surgery with weight-loss medications in treating obesity?
  - ***Prompt:*** How would you compare surgery and medication in terms of effectiveness and patient outcomes?
- What factors do you think influence a patient’s decision between surgery, medication, or lifestyle interventions?

**Closing**

Notable interactions with patients

- Can you share a story about a patient you have supported who left a lasting positive impression on you?

Demographics:

- Do you work as a ________ [**profession**] in primary or secondary care?
- May I ask how old you are?
- What is your gender identity?

***We were interested in your experiences with people living with obesity and patients undergoing bariatric surgery.***

- How did you find the interview?

***Thank the participant for their time and contribution.***
