# Supplementary material for: Attitudes and experiences of lifestyle healthcare professionals in the care of metabolic and bariatric surgery patients in the Netherlands
Source: Obes Pillars. 2026 Mar 25;18:100261. doi: 10.1016/j.obpill.2026.100261 (PMC13054046; doi:10.1016/j.obpill.2026.100261)
Supplement: Multimedia component 2 [file mmc2.docx]

**Appendix B - Supplemental Table 1**. Checklist Consolidated Criteria for Reporting Qualitative Research (COREQ) for reporting qualitative research

| **Domain 1: Research team and reflexivity** | |
| --- | --- |
| **Personal characteristics** | |
| 1. **Interviewer/facilitator**: Which author/s conducted the interview or focus group? | First author and student assistant |
| 2. **Credentials:** What were the researcher’s credentials? E.g. PhD, MD | Author 1: PhD researcher in Social Sciences.  Author 2: Associate Professor of Strategic Communication  Author 3: Personal Professor of Participatory Community Health Promotion.  Author 4: Surgeon specializing in Upper GI and metabolic bariatric surgery; Special Professor of Nutrition and Obesity Treatment.  Author 5: Associate Professor of Health and Society, expertise psychosocial determinants and outcomes of health behavior. |
| 3. **Occupation**: What was their occupation at the time of the study? | Author 1: Lecturer and a PhD student at a university.  Author 2: Associate Professor at a university. Expertise in perceptions, beliefs, and emotions shaping human behavior.  Author 3: Personal Professor at a university. Expert in lifestyle, social, and environmental determinants of health, using participatory action research and whole-systems approaches  Author 4: Surgeon with expertise in bariatric surgery, reflux (acid) surgery, and hiatal hernia repair; And Special professor at a university with research focused on minimally invasive treatment of esophageal and gastric disorders and obesity.  Author 5: Associate Professor working at a university. Expertise psychosocial determinants and outcomes of health behavior. |
| 4. **Gender:** Was the researcher male or female? | Author 1, 3, & 5: Female  Author 2 & 4: Male |
| 5. **Experience and training:** What experience or training did the researcher have? | All authors have experience with qualitative research and interviewing, as well as relevant expertise in the study topic. |
| **Relationship with participants** |  |
| 6. **Relationship established**: Was a relationship established prior to study commencement? | Participants had no prior contact with the interviewers. None of them knew about the researchers before beginning the study. Participants were informed by email and at the start of the interview about the study and their rights before the interviews. |
| 7. **Participant knowledge of the interviewer**: What did the participants know about the researcher? e.g. personal goals, reasons for doing the research | Participants were informed about the researcher's background and purpose of the study, and ethical rights (e.g. voluntary, right to withdraw without reason, confidentiality etc.) |
| 8. **Interviewer characteristics:** What characteristics were reported about the inter viewer/facilitator? e.g. Bias, assumptions, reasons and interests in the research topic | Reflexivity was maintained by following a semi-structured interview protocol to ensure neutrality, engaging in regular peer-debriefing and team discussions, and discussing interpretations within the research team, and sharing interpretations with research participants to validate findings. |
| **Domain 2: Study design** |  |
| **Theoretical framework** |  |
| 9. **Methodological orientation and Theory:**  What methodological orientation was stated to underpin the study? e.g. grounded theory, discourse analysis, ethnography, phenomenology, content analysis | The study explored attitudes - referring to beliefs, feelings, views, and opinions - toward MBS and its patients (Eagly & Chaiken, 1993), as well as experiences in providing care and support to this patient group. A qualitative descriptive approach was used to investigate these attitudes and experiences and identify challenges or gaps in healthcare practice, employing semi-structured interviews for data collection. Data were analyzed using thematic analysis following Braun and Clarke’s framework. |
| **Participant selection** |  |
| 10. **Sampling:** How were participants selected? e.g. purposive, convenience, consecutive, snowball | A purposive sampling approach was used. Participants were eligible if they were registered lifestyle healthcare professionals (LHCPs)—including dietitians, nutritionists, lifestyle coaches, physiotherapists, or other lifestyle-focused professionals—and had experience caring for patients with obesity and MBS in the pre- or postoperative phase. |
| 11. **Method of approach**: How were participants approached? e.g. face-to-face, telephone, mail, email | Participants were recruited via email (367 invitations), professional networks, social media (e.g., LinkedIn), and snowball sampling, yielding 38 responses. |
| 12. Sample size: How many participants were in the study? | 22 participants |
| 13. **Non-participation:** How many people refused to participate or dropped out? Reasons? | Of 38 interested respondents, two were ineligible, eight could not participate due to time constraints, and four did not respond to follow-up, resulting in 24 interviews. Two participants had only general obesity experience, leaving 22 included in the study. |
| 14. **Setting of data collection**: Where was the data collected? e.g. home, clinic, workplace | All interviews were conducted either online via Microsoft Teams. |
| 15. **Presence of non-participants**: Was anyone else present besides the participants and researchers? | Only the participant and the interviewer were present. |
| 16. **Description of sample**: What are the important characteristics of the sample? e.g. demographic data, date | The sample included 13 dietitians, 8 lifestyle coaches, and 1 physiotherapist/manual therapist. Most participants (n = 18) worked in primary care, and 4 in secondary care. Participant characteristics are further detailed in Table 1. |
| **Data collection** |  |
| 17**. Interview guide:** Were questions, prompts, guides provided by the authors? Was it pilot tested? | A semi-structured interview guide was developed, with questions reordered after the first interview for better flow without changing their wording. |
| 18. **Repeat interviews**: Were repeat interviews carried out? If yes, how many? | No |
| 19. **Audio/visual recording:** Did the research use audio or visual recording to collect the data? | Interviews were audio-recorded and transcribed with participants’ consent. |
| 20. **Field notes:** Were field notes made during and/or after the interview or focus group? | No |
| 21. **Duration:** What was the duration of the interviews or focus group? | Interviews lasted an average of 43 minutes, ranging from 30 to 65 minutes. |
| 22. **Data saturation**: Was data saturation discussed? | Data saturation was discussed with the research team and is further addressed under Strengths and Limitations. |
| 23. **Transcripts returned:** Were transcripts returned to participants for comment and/or correction? | No |
| **Domain 3: analysis and findings** |  |
| **Data analysis** |  |
| 24. **Number of data coders**: How many data coders coded the data? | All transcripts were systematically coded by the first author, with six transcripts co-coded by the research team to validate the findings. |
| 25. **Description of the coding tree:** Did authors provide a description of the coding tree? | A codebook was initially developed deductively from the interview guide, with additional inductive codes added to capture unanticipated insights. It was collaboratively reviewed and refined by the research team to ensure coherence and consistency. |
| 26. **Derivation of themes:** Were themes identified in advance or derived from the data? | Themes were identified inductively from the data and deductively based on the interview guide.. |
| 27. **Software:** What software, if applicable, was used to manage the data? | Atlas.ti version 24 |
| 28. **Participant checking**: Did participants provide feedback on the findings? | Findings were shared with participants via email and online through Teams, with three participants responding to confirm the results. |
| **Reporting** |  |
| 29. **Quotations presented**: Were participant quotations presented to illustrate the themes/findings? Was each quotation identified? e.g. participant number | Findings are presented in the Results section and Tables 2–5, with each quote identified by participant number. |
| 30. **Data and findings consistent**: Was there consistency between the data presented and the findings? | The data presented in the study and the results that emerge from them are consistent. |
| 31. **Clarity of major themes:** Were major themes clearly presented in the findings? | Major themes are reported in the Results section under distinct headings. |
| 32. **Clarity of minor themes**: Is there a description of diverse cases or discussion of minor themes? | Minor subthemes are reported with the major themes and supported by participant quotes. |

Tong, A., Sainsbury, P., & Craig, J. (2007). Consolidated criteria for reporting qualitative research (COREQ): a 32-item checklist for interviews and focus groups. *International journal for quality in health care : journal of the International Society for Quality in Health Care*, *19*(6), 349–357. https://doi.org/10.1093/intqhc/mzm042
